# Urinary Test for Pregnancy

**Published:** 1915-11

**Authors:** 


**Urinary Test for Pregnancy.** Chas. H. Walker and Frederick Klein of N. Y., Am. Med., Sept. The general principle of the reaction seems to be a state of suboxidation, most of the oxygen absorbed being taken up by newly formed cells. To 1 gram of ferrous sulphate, is added 2 c.c. of water and after solution, 10-15 c.c. or 3% hydrogen peroxid solution. With the evolution of oxygen and ozone, a reddish brown color develops (ferris hydroxid?). After effervescence has subsided, and equal volume of glycerin of s.g. 1250 is added. 1-2 c.c. of the reagent is added to 4 c.c. of urine and then an equal volume of strong HCL, s.g. 1190. A normal, nonpregnant urine should be used as a control, pregnant urine giving a canary yellow color, others a reddish-brown or dark orange color. (Note: Having entered upon medicine at about the time of the explosion of the kyestin fallacy, we are decidedly sceptic as to any such reaction to determine pregnancy. However, the test is worth trying and the editor will gladly co-operate with members of the profession to determine its reliability. To insure impartial results, it is suggested that controls, male and female should accompany the urine of pregnant patients, designated in such a way that the experiment has no knowledge of the condition in advance).

The authors also suggest the following confirmatory test. To 25 c.c. of glacial acetic acid, is added 0.8 gram of benzidin and ½ c.c. of a 5% aqueous solution of selenous acid, and heated till a green solution is obtained. ½ c.c. of this reagent is added to 1-2 c.c. of urine, normal urine giving a heavy precipitate, pregnant urine none. Dark or old urine should be previously filtered through animal charcoal or a mixture of charcoal and kaolin. The same tests are applied in the case of cancer and syphilis, the urine first being precipitated with lead acetate and filtered.

